# UK Dietary Policy for the Prevention of Cardiovascular Disease

**DOI:** 10.3390/healthcare5010009

**Published:** 2017-02-20

**Authors:** Louis Levy, Alison Tedstone

**Affiliations:** 1 Diet & Obesity, Public Health England, London SE1 6LH, UK; alison.tedstone@phe.gov.uk; 2 Faculty of Health & Social Care, University of Chester, Chester CH1 1SL, UK

**Keywords:** dietary recommendations, CVD prevention, obesity prevention, Eatwell Guide

## Abstract

Nutrition advice is devolved within each of the four UK countries, but share a common evidence base provided through the Scientific Advisory Committee on Nutrition (SACN). Current UK dietary recommendations to prevent cardiovascular disease (CVD) is based upon recommendations from SACN and its predecessor committee. Dietary advice in the UK has recently been revised in relation to intakes of free sugar and fibre. This paper highlights current UK recommendations for the prevention of CVD, in particular related to energy intake, saturated fat, free sugars, salt, fruit, vegetables, oily fish and fibre. It describes how this advice is promulgated including the refresh of the Eatwell Guide and wider action that will impact on CVD.

## 1. Introduction

Cardiovascular disease (CVD) affects around seven million people in the UK and is a significant cause of disability and death [1], responsible for 27% of all deaths (126,682) in England in 2014 [2]. A significant proportion of these deaths are premature: 25% in men and 17% in women under the age of 75. CVD accounts for more than 15% of total disability adjusted life years in England [3].

Overweight and obesity along with a poor diet, smoking, being physically inactive, and drinking too much alcohol are all risk factors for CVD; and such lifestyle factors are likely to be more ubiquitous in lower socioeconomic groups in the UK [4]. Mortality rates from CVD in people under 75 years are 105 per 100,000 in the most deprived decile compared with 59 per 100,000 in the least deprived decile in 2012–2014 [5].

This paper highlights current UK recommendations for the prevention of CVD and action to improve population diet and thus population health.

## 2. UK Government Recommendations

The Scientific Advisory Committee on Nutrition (SACN), a group of independent experts, advise the UK government in England, Scotland, Wales and Northern Ireland, government departments and Public Health England on nutrition issues. SACN and its predecessor group, the Committee on Medical Aspects of Nutrition Policy (COMA, prior to 2000) assess the impact of diet on health and make recommendations for dietary intake. The conclusions from SACN considerations are translated by Public Health England and relevant government departments across the UK into national recommendations and public facing advice.

SACN uses a framework approach, when developing its reviews of the available evidence. This ‘hierarchy of evidence’ [6], includes consideration of strength of evidence according to study design. Typically, randomised controlled trials (RCTs) are apportioned most weight with observations (non-intervention) studies being accorded less weight owing to the increased potential for bias, confounding and reverse causality. SACN’s approach however, recognises that it may not be feasible or ethical to conduct RCTs in every situation and evidence from well conducted prospective or other studies are also considered to inform SACN’s conclusions. SACN also utilises high quality systematic reviews to capture all the evidence available on a subject when this is relevant to answer a specific question. SACN procedures provide for a public consultation on draft reports to ensure opportunities for further relevant, high quality, evidence to be provided and comments from a wide range of stakeholders to be considered before it reaches and publishes its final reports.

The basis for UK dietary recommendations and cardiovascular health have been previously discussed [7]. This included evidence for reducing plasma total and Low Density Lipoprotein (LDL) cholesterol [8,9], salt [10] and weight through dietary energy, saturated fat, salt, fibre [8], fish [11] and fruit and vegetables [12].

Since the recommendations for cardiovascular health and diet were adopted further data on dietary intake and urinary sodium in the UK alongside the SACN report on carbohydrates and health have been published.

In the UK, dietary reference values have previously been provided for ‘non-milk extrinsic sugars’ that is sugars not contained within the cellular structure of a food except lactose in milk and milk products. The SACN Carbohydrates and Health report reinforced the need for reductions in sugar consumption in relation to energy intake and thus weight gain, setting a new maximum recommendation at 5% of energy for a newly defined free sugar intake [13]. In this definition, free sugars comprise all monosaccharides and disaccharides added to foods by the manufacturer, cook or consumer, plus sugars naturally present in honey, syrups and unsweetened fruit juices. Lactose when naturally present in milk and milk products is excluded from this definition.

UK adults are currently consuming an average 12.5% of food energy from non-milk extrinsic sugars (NMES) [14]. This is more than double the new recommendation assuming that the new definition of free sugar is similar to that for NMES in the UK’s National Diet and Nutrition Survey (NDNS); a continuous, cross-sectional survey designed as a rolling programme to collect detailed, quantitative information on the food consumption, nutrient intake and nutritional status of the general population aged 1.5 years and over living in private households in the UK, covering a representative sample of around 1000 people per year. The contribution of sugar sweetened beverages to sugar intakes remain of concern particularly for children and young people.

SACN concluded that increased intakes of total dietary fibre, and particularly cereal fibre and wholegrains, was strongly associated with a lower risk of cardio-metabolic health outcomes [13]. They also concluded that higher intake of oat bran and isolated β-glucans leads to lower total cholesterol, LDL cholesterol and triacylglycerol concentrations and lower blood pressure. As a result of their assessment of the evidence SACN recommended changes to the definition and recommended values, increasing the recommendation to 30 g of fibre a day measured using the Association of Official Analytical Chemists method for total dietary fibre analysis (AOAC) compared to the previous recommendation of 18 g of non-starch polysaccharide a day (equivalent to 23 g AOAC fibre). Mean intakes of non-starch polysaccharide remain well below the dietary recommendation at a population average intake of 18 g per day for adults aged 19 years and over (14 g/day in adults aged 19–64 years, 13.4 g/day in adults aged 65+ years [14].

Salt intakes in UK adults fell by a mean daily estimate of 0.9 g/day between 2005/2006 and 2014—a relative reduction of 11% achieving a mean estimated salt intake of 8.0 g a day [15]. Approximately two thirds of results from participants in England exceeded the recommended maximum of 6 g of salt a day with 32.4% of men and 26.9% of women recording high blood pressure [16]. Reducing UK population salt intakes by 1 g is estimated to prevent 4147 premature deaths and save the NHS £288 million each year [17].

Seventy-four percent of UK 11 to 18 year olds and 68% of adult aged 19–64 years have intakes of saturated fat that exceed the Dietary Reference Value of 11% food energy [18]. Around two thirds of UK men and women have blood cholesterol greater than the recommended 5 mmol/L. Estimates suggest that if the UK population were to reduce saturated fat intake to recommended levels 2600 premature deaths would be averted each year [19]. While several commentators suggest consumption of high fat, low carbohydrate diets would benefit population health [20,21] this approach would not be compatible with the recommendation from the SACN Carbohydrates and Health report [13] which concluded that carbohydrates should remain about half of total energy intake. A SACN working group has been established to consider evidence on population recommendations for saturated fat. A draft report for consultation is anticipated in late 2017.

Fruit and vegetable consumption by UK adults aged 19 to 64 years achieves an average value of 4.0 portions per day while adults aged 65 years and over consumed 4.2 portions per day. Twenty-five per cent of men aged 19 to 64 years, 28% of women aged 19 to 64 years, 34% of men aged 65 years and over and 35% of women aged 65 years and over met the 5-A-Day recommendation[14]. Such intakes therefore fail to meet recommendations associated with lower incidence of cardiovascular disease (that is consumption of more than 400 g a day [12]; equivalent to at least 5 portions as defined by the UK government). Furthermore, reduced risk of ischaemic heart disease mortality was associated with higher intake of fruit and vegetables at levels commensurate with the recommended intakes [22].

Mean oily fish consumption in the UK was equivalent to 14–28 grams per week in children and 56–84 grams per week in adults well below the 140 g portion recommended [18].

These data indicate that there remain considerable movements for UK diet to achieve improvements in cardiovascular health. Current UK dietary macronutrient and major food recommendations for UK are summarised in Table 1. Information on UK dietary recommendations by age and gender are available in PHE’s Government Dietary Recommendations publication [23].

## 3. Eatwell Guide

In the UK, healthy eating advice is translated into a food model visual to help the public understand and follow a diet consistent with health. In 1994 this was depicted as the *Balance of Good Health* [24,25,26] based upon a model average diet developed as part of the COMA Nutritional Aspects of Cardiovascular Disease report [8] using household food purchasing data from the 1992 National Food Survey. This was updated in 2006 to the eatwell plate [27] when health professionals began to express an interest in seeing an updated, more appealing visual. Consumer research was conducted to understand attitudinal and behavioural responses to differing visual representations of healthy eating and the context in which people viewed these. Since government dietary advice had not changed since the development of the Balance of Good Health, the proportions associated with different food category groups was not reviewed.

Given the likely changes to sugar and fibre recommendations that were emerging in 2015 when SACN published its draft report on Carbohydrates and Health, PHE committed to reviewing healthy eating messages—including the eatwell plate. PHE convened an external reference group to advise on methodology for this review [28]. Given the potential changes in dietary recommendations several approaches to updating the model were considered. Linear programming was identified as the most robust and objective approach. This modeling approach uses a mathematical function that measures the divergence of the modelled scenario from the diet currently consumed; resulting in a scenario that has the fewest number of changes to achieve dietary recommendation and is described elsewhere [29,30]. Modelling utilised the most recent NDNS data at the time of the modelling [18] and most recent data on food composition [31] with macronutrient and food recommendations for adults as the outcome variables (Table 2).

Alongside this modelling, qualitative work took place across the UK involving 152 individual in-depth interviews with individuals (60 from higher (BC1) socioeconomic groups, 92 from lower (C2DE) socioeconomic groups) in phase 1 and 80 individual depth interviews (20 BC1, 61 C2DE) in phase 2. Interviewees included Caucasian, Afro-Caribbean, African, Chinese, South Asian and mixed race individuals. This research assessed the public understanding of the eatwell plate and approaches to improving understanding, meaning and opportunities to improve the communication of healthy eating messaging. Research was undertaken in two phases with the second phase building on the learning form the first phase and utilisng early outcomes from linear programming to change the balance of food groups within the visual [29].

Overall, the eatwell plate model of a circular visual was well recognised and understood by the public. While participants recognised they did not meet the recommendations in the visual they were able to interpret their own diet relative to this and identify how they could improve their diet to meet healthy eating recommendations. The use of drawn images rather than photographs (as previously used in the eatwell plate) was preferred for recognition, clarity and educational potential while photographs of food was seen as being too aspirational. Based on feedback from phase 1 research changes to the names of food group categories were positively received as were the additional messages provided outside individual food group segments.

The greatest difference in the visual image related to the separation of ‘foods to be consumed less often and in small amounts’ from a small segment allocated to ‘oils and fats’. The separation of these foods and movement outside the main image were considered to convey better understanding of the role of these foods in the diet. Participants welcomed the inclusion of hydration messages and the advice to reduce sugary drinks within this. The inclusion of adult energy intake recommendations as part of the border of the image was seen to be useful approach of communicating benchmark energy intakes.

**Figure 1:**
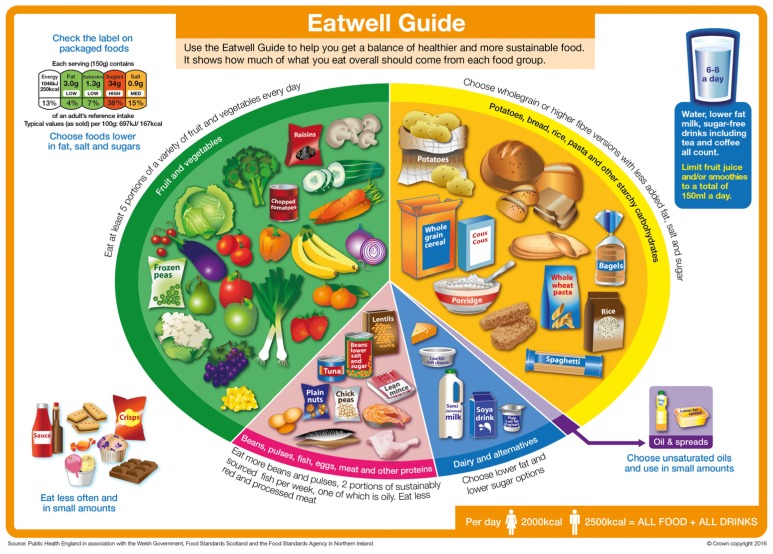
The Eatwell Guide. The UK National Food Model Updated from the eatwell plate in March 2016.

Following the finalisation of modelling and the consumer research, PHE revised and launched the Eatwell Guide in March 2016 (Figure 1).

Helping the UK population achieve dietary recommendations is not going to be realised through a revised food model alone. Action arising by government working with industry in the UK to reduce salt in manufactured products alongside a government salt awareness campaign has been associated with an 11% reduction in salt intake since 2009 [15]. Similar activity related to sugar, saturated fat and portion size would support reductions in energy intake that would be anticipated to impact on weight and associated ill health, including that for CVD.

Sugar reduction evidence into action [32] considered a range of evidence to assess approaches to reducing sugar intake in the UK. This mixed method research concluded that no single approach would be sufficient to reduce obesity in the UK, however, a multifaceted approach taking a whole system approach had the potential for impact. Action, which if enacted together would likely reduce UK, sugar intakes included reducing and rebalancing price promotions in all retail outlets, reducing marketing and advertising of high sugar food and drink across all media, setting a clear definition for high sugar foods, Introducing a broad sugar reduction programme in everyday food and drink, introducing a minimum 10%–20% tax or levy on high sugar products e.g. sugary soft drinks, adopting the government buying standards for food and catering services more widely across the public and private sectors, ensuring delivery of accredited training in diet and health to the catering, fitness and leisure industry workforce and raising awareness of sugar intakes to the public, workforce, employers and food industry.

The UK Government’s Childhood Obesity Plan was published in August 2016 [33] and included some of the areas of work highlighted in Sugar reduction evidence into action. While chiefly addressing obesity, the potential impact on weight, together with other actions on CVD risk factors are likely to improve CVD outcomes in the UK. Activity alongside that related to diet and obesity addressing adult CVD include work on alcohol, tobacco, physical activity and pollution [34].

## 4. Conclusions

Government advice in the UK is based upon review of the evidence base. Dietary advice in relation to cardiovascular disease takes account of energy, saturated fat, sugar, salt, fruits, vegetables, fibre and oily fish. The UK’s Scientific Advisory Committee on Nutrition publishes evidence reviews in these, and other areas; most recently their report on Carbohydrate and Health. Based on this advice the UK updated its national plate model represented as the Eatwell Guide. Moving the population to a diet consistent with the Eatwell Guide will help people meet UK dietary recommendations and improve health, including cardiovascular health resulting in longer and healthier lives.

## Figures and Tables

**Table 1:** UK macronutrient and food recommendations for cardiovascular health.

	Dietary Recommendation
Nutrients	
Energy	2000 kcal (8.4 MJ) for women; 2500 kcal (10.4 MJ) for men
Carbohydrates	≥50% of total energy
Free Sugars	≤5% food energy
Fat	≤35% food energy
Saturated Fat	≤11% food energy
Salt	≤6 g/2363 mg sodium
Fibre (AOAC)	30 g
Foods	
Fruits and Vegetables	At least 5 portions of a variety each day
Fish	At least 2 portions a week, one of which should be oily

**Table 2:** Mathematical constraint factors (used to ensure dietary recommendations met in solved linear programming model) utilised to develop the visual reflecting government dietary recommendations.

	Dietary Recommendation	Constraint Factors Used in Linear Programming Model (Exclusing Alcohol)
Nutrients		
Energy	2250 kcal (9414 MJ) ^1^	No increase ^4^
Carbohydrates	≥50% of total energy	≥50% of food energy
Free sugars	≤5% food energy	≤5% food energy
Fat	≤35% food energy	≤35% food energy
Saturated fat	≤11% food energy	≤11% food energy
Protein	Approx. 15% food energy	≥14.5 & ≤15.5% of energy
Salt	≤6 g/2363 mg sodium	≤6 g/2363 mg sodium
Fibre (AOAC) ^2^	30 g	≥30 g
Foods		
Fruits and vegetables ^3^	At least 5 portions of a variety each day	≥5 portions a day
Fish	At least 2 portions a week, one of which should be oily	≥2 portions (2 × 140 g) a week, one of which should be oily
Red and processed meat	High consumers should reduce their intake to the average of the population (70 g)	≤70 g/day

AOAC: Association of Official Analytical Chemists method for total dietary fibre analysis; ^1^ Assumes mixed population average; ^2^ Equivalent 18 g non-starch polysaccharide fibre; ^3^ Includes a maximum of: 1 portion of juice (from fruit/vegetable juice or that in a smoothie); 1 portion of beans; (Portion sizes: 30 g dried fruit; combined total of 150 mL of fruit and/or vegetable juice and/or smoothie; 80 g all other fruits and vegetables); ^4^ energy from NDNS intakes (weighted average for adults equivalent to 1711 kals (7159 MJ).
